# RC-GNN: A predictive model of enzyme-reaction pairs

**DOI:** 10.1101/2025.06.22.660952

**Published:** 2025-06-27

**Authors:** Stefan C. Pate, Eric H. Wang, Linda J. Broadbelt, Keith E.J. Tyo

**Affiliations:** 1 Department of Chemical and Biological Engineering, Northwestern University, Evanston, IL, USA; 2 Center for Synthetic Biology, Northwestern University, Evanston, IL, USA; 3 Interdisciplinary Biological Sciences Graduate Program, Northwestern University, Evanston, IL, USA; 4 Piton Therapeutics

## Abstract

Uncharacterized functions of enzymes represent untapped opportunity to develop therapeutics, unlock the sustainable synthesis of materials, and understand the evolution of life-sustaining metabolic networks. Enzymes and *de novo* reactions (i.e., non-native, promiscuous reactions), generated by protein language models and computer-aided synthesis tools, respectively, make up a large part of this opportunity. Given the technical complexity of high-throughput enzymatic activity screens, predictive models are needed that can pre-screen *de novo* enzyme-reaction pairs *in silico*. We present Reaction-Center Graph Neural Network, (RC-GNN) a model capable of predicting whether an enzyme, represented by an amino acid sequence, can significantly catalyze a given reaction, represented by its full set of reactants and products. We explicitly evaluated RC-GNN’s generalization to *de novo* queries. In the most difficult conditions tested, where difficulty is measured by the level of dissimilarity between training and test data points, the model achieves 78.0% and 94.8% accuracy when reaction and enzyme similarity were respectively controlled. The ability to successfully make predictions on enzymes and reactions distinct from those used during training make RC-GNN especially useful for both metabolic engineers and evolutionary biologists who need to reason about uncharacterized enzymatic reactions.

## Introduction

A complete mapping of enzyme sequences to the reactions they catalyze would enable progress across multiple fields: pharmaceutics, biomanufacturing^1,2^, physiology, and evolution^3–7^. Over decades, researchers have characterized the function of thousands of enzymes, namely the canonical reactions that they catalyze. Nevertheless, it is widely believed that our collective knowledge is far from complete. In large part, the missing pieces are secondary functions, also known as promiscuous activities of enzymes, or the underground metabolism^8^. Typically, these activities are less efficient than the primary function but far more efficient than the uncatalyzed reaction. To an evolutionary biologist, they represent both vestiges of evolutionary history and sources of evolvability required for future phenotypic change. To a biotechnologist, they represent both a challenge and an opportunity. Secondary functions required for a synthesis pathway can be elicited with protein engineering. On the other hand, they are sources of unanticipated adaptations to a metabolic engineer’s purposeful genetic perturbation.

To put the experimental challenges associated with mapping enzyme-reaction pairs into context, testing all possible enzyme-reaction pairs from the dataset studied in this work would require hundreds of millions of experiments. Furthermore, this does not include *de novo* enzymes and *de novo* reactions, as one considers in computer-aided biosynthesis. At the same time, throughput of a systematic screen of enzymatic functions is limited by a lack of detection methods applicable to such a wide range of reactants and products^8^.

Given this challenge, a critical question is what experiments should be prioritized in order to most efficiently increase our knowledge of enzymatic function. While theoretical^9^ and heuristic understanding of enzymatic reaction mechanisms can greatly constrain this search, the range of structures (of both substrates and enzymes) and functions is great, and the relationship between them is rich. Machine learning (ML) is an appropriate tool to learn such a complicated relationship from data. An ML model can inform decisions on experiment prioritization, and its parameters can be updated with the experimental results to close the loop of semi-automated exploration.

Such a model must accept two inputs, an enzyme and a reaction, and score the hypothesis that the former catalyzes the latter. Previous work has formulated several related tasks to the one just described. One group of models outputs Enzyme Commission (EC) numbers given an amino acid sequence or reaction^10–16^. Predicting EC numbers is useful but limited in that it is not a precise description of enzymatic function^17–19^. A second group of tools looks up enzymes empirically associated with characterized reactions similar to a query reaction or substrate^20–23^. These models are limited to the task of finding enzymes already associated with known reactions and mostly utilize non-differentiable methods to perform the lookup. A third group of models scores the interaction between the enzyme and a putative substrate^24,25^. While it is useful to know if a substrate and enzyme interact, this fact alone leaves ambiguity around the transformation that the substrate undergoes. For example, pyruvate may undergo several reactions including reduction to lactate (EC: 1.1.1.28), decarboxylation to acetaldehyde and carbon dioxide (EC: 4.1.1.1), or carboligation to acetolactate (EC: 2.2.1.6). A primary task of computer-aided biosynthesis is to predict an enzyme to catalyze a reaction^2^, and the design of an experiment to test such a prediction includes at a minimum an amino acid sequence and the chemical structures of all reactants and products.

Here we developed several models which vary in the architecture of their reaction encoder and use ESM-1b^26^ to encode the amino acid sequence of the enzymes. All of them reliably predict whether an enzyme catalyzes a reaction. One among them, the reaction center (RC) aggregated encoder, which uses a chemistry-inspired form of graph augmentation to encode reactions, generalizes well on highly dissimilar queries. To demonstrate generalization to highly dissimilar queries, we quantify data point similarity with both protein and reaction-based pairwise similarity metrics. We use these metrics to perform stratified similarity split on our dataset, which enables performance evaluation as a function of (dis)similarity. The performance of the RC aggregated model is most differentiated in the most difficult task, in which models are challenged to infer enzyme pairs for highly dissimilar reactions than were available in the training set. RC aggregated achieved 78.0% accuracy compared to the next best model with 68.8% accuracy. In the most difficult protein-based split, RC aggregated and the next best model each achieve an accuracy of 94.8%. Taken together, our results suggest the developed model will perform well on *de novo* reactions and enzymes.

## Results

### Preparing a dataset of enzyme-reaction pairs

We acquired experimentally validated enzyme-reaction pairs from UniProt, which features cross-referenced enzymatic reactions from the Rhea database^27,28^. We restricted our dataset to pairs with direct evidence, not inferred from homology. A lack of true negative samples is a common issue in binary classification tasks and was true in our case. In lieu of true negatives, practitioners use one of several strategies to sample negative data points^29,30^. Two common approaches are global selection from all unobserved pairs, and the use of an auxiliary similarity metric to bias negative samples to be more or less similar to observed pairs^29^. Prior work on enzyme function prediction has used auxiliary similarity metrics to bias negative samples in both directions^24,25^. We instead opt for global selection of negatives, relying only on a basic assumption that the underlying adjacency matrix of enzymes and reactions is sparse, and avoiding any assumptions about the structure-function relationship, which can exhibit discontinuities as discussed in other domains of cheminformatics^31^. The adjacency matrix of the observed enzyme-reaction pairs has a density of 0.03%. The density of the true adjacency matrix being inferred is unknown^8^; however, assuming current knowledge covers at least 10% of enzyme promiscuity, the probability of misassigning true negatives is less than 3%.

A major focus of this work is to demonstrate model generalization to *de novo* enzymes and reactions. To this end, we utilized protein and reaction similarity metrics to precisely quantify (dis)similarity. This approach has been used in prior ML tasks involving proteins to avoid overly optimistic estimates of generalization error^32^. To address this issue on the reaction side, we developed reaction center maximum common subgraph (RCMCS). Analogously to global sequence identity (GSI), which we use to measure pairwise protein similarity, RCMCS is based on an alignment of reaction graphs. These similarity metrics were used in a stratified similarity split procedure to ensure that models were evaluated on test data points spanning a range of dissimilarity from the training set. See Methods for details.

### Constructing a model to map enzymes and reactions into a joint embedding space

**Figure 1:**
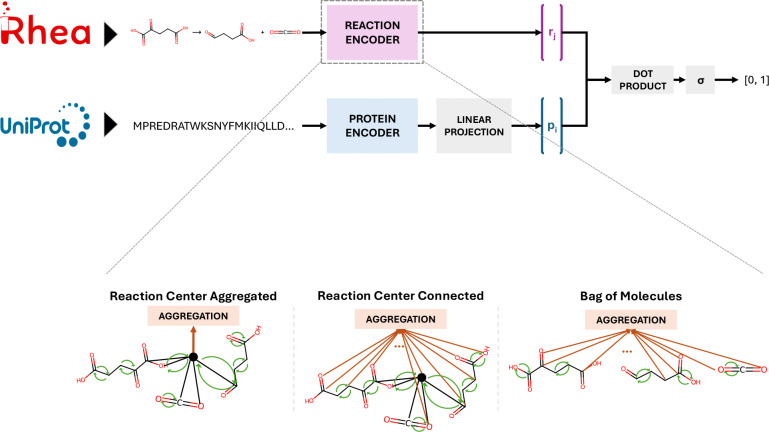
Overview of the model architecture. (Top) All models take as input a pair of reaction, encoded as a SMILES string, and a protein, encoded as an amino acid sequence. Each item in the pair is transformed into a vector representation by an encoder. Finally, the pair of vectors is converted into a score, which is a function of their dot product. (Bottom) Several variants of reaction encoders were compared. Bag of molecules (right) represents a reaction as the mean of all atom embeddings following several rounds of message passing. RC aggregation (left) represents a reaction with the embedding of a virtual node which has been connected to RC atoms via virtual edges. RC connected (center) also introduces a virtual node connected to the RC but represents a reaction as the mean of all atom embeddings as in bag of molecules.

To predict the catalysis of a reaction by an enzyme we took the approach of learning representations of both. We develop models that map each item in the pair to the same vector space. With this, we can score whether the enzyme in a pair would catalyze the reaction by taking a dot product between the two items’ vector embeddings. This architecture of model requires two encoders (Fig. 1a). The protein encoder consists of the ESM-1b transformer model which maps an amino acid sequence to a 1280-dimensional vector^26^. The ESM vector embedding is then linearly transformed with a matrix learned from the current task. The architecture of the reaction encoder, which maps the SMILES^33^ representation of a reaction to a vector embedding, is a major focus of the present work.

Graph Neural Networks (GNN) have been used to generate learned molecular embeddings^34^, and the Chemprop package offers a suite of GNN functionality bespoke to cheminformatics^35^. Building off this prior work, we developed and compared three GNN-based reaction encoders (Fig. 1b and Table 1). The first, bag of molecules, represents a reaction with the mean embedding of atoms across all reactants and products, each of which is generated after a number of message passing steps. The RC aggregated encoder augments the graph of reactants and products with a virtual node as done previously in molecular property prediction^36^. In our case, we augmented the graph by connecting a virtual node to the RC atoms by virtual edges. Though initially stateless, the virtual node inherits a combination of atom and bond features through message passing. This combination includes features within *k* bonds of the RC where *k* is one less than the number of message passings. The RC connected encoder is a hybrid between the former two. Like RC aggregated, it augments the reaction graph with a virtual node, but the reaction is represented as the mean atom embedding like in the bag of molecules encoder.

Additionally, we compared these three GNN encoders with a condensed graph of reaction (CGR) encoder^37^ implemented in Chemprop, and a linear combination of reactant and product extended-connectivity fingerprints (ECFP)^38^ also referred to as Morgan fingerprints. For a summary of reaction encoders, see Table 1. Like the GNNs, the Morgan fingerprint encoder works by iterative message passing, but without a learnable weight matrix combining the messages. On each side of the reaction, substrate fingerprints were summed. Then the absolute difference between the summed fingerprints of each side was taken as the reaction fingerprint. Finally, the reaction fingerprint was transformed linearly with a learned matrix to match the dimension of the enzyme embedding.

### Successfully predicting unseen enzyme-reaction pairs

We estimated generalization error on test sets generated using a stratified similarity split technique. This splitting technique ensures that the maximum pairwise similarity of a test data point to train data points varies over a wide range. In other words, the test set consists of a subset of data points with low similarity to the training set, a subset with high similarity to the training set, and so on for several intermediate levels of similarity. Since our data points are pairs of enzymes and reactions, we carry out the stratified similarity splitting procedure twice, using RCMCS and GSI for reactions and enzymes, respectively. See Methods for details on stratified similarity split. See Fig. S1 for the distribution of pairwise similarities over the whole data set and in our test set, relative to the training set.

**Figure 2:**
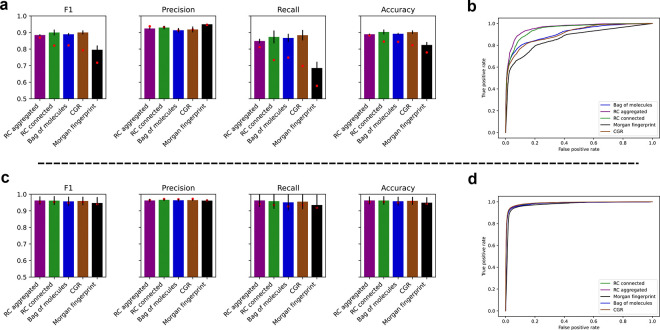
Models successfully predict enzyme-reaction pairs. (a) Classification performance metrics plotted for each type of model. Red points show test performance. Bar graphs show mean performance on validation data during cross validation. Error bars are standard deviation. (b) ROC curves plotted for each type of model using test data. In both (a) and (b), stratified similarity split with RCMCS as the similarity metric was used to split data. (c) and (d) Same as (a) and (b), respectively, but using GSI as the similarity metric in stratified similarity split.

We find in general that splitting based on pairwise reaction similarity, RCMCS, leads to a more challenging task than GSI-based splitting. While all models perform above chance at labeling unseen enzyme-reaction pairs as positive or negative, the GNN models outperform the Morgan fingerprint benchmark on reaction-based stratified similarity splits (Fig. 2a and 2b). This difference is especially pronounced in the recall score. One can see that the RC aggregated model generalizes better to the test set, shown as red points in Fig. 2, though this and all other differences are muted in the protein-based splits (Fig. 2c and 2d). Such robustness to protein dissimilarity has been observed before^24^. Although we were motivated to find the point at which performance would decline, we were limited to a lower bound of 40% GSI beyond which the dataset collapsed into a single cluster, precluding stratified similarity splitting.

### Chemistry-inspired graph augmentation leads to robust model performance

We sought to understand the relationship between reaction similarity and the difficulty of predicting enzyme function by leveraging our stratified similarity splitting technique. We hypothesized that a model would perform worse where query reactions look quite different from the training set. This is consistent with the general notion that machine learning models excel at interpolation within a distribution of data but not extrapolation beyond it^39^. Alternatively, it could be true that the task becomes difficult in the other extreme where query reactions are quite similar, but with subtle differences that affect function to an outsized extent^31^.

**Figure 3:**
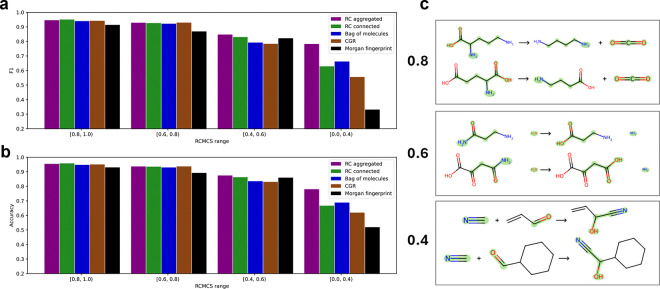
RC aggregated model performs best on highly dissimilar reactions. (a) Test set F1 score plotted against maximum RCMCS similarity between test data points and train data points. (b) Same as (a) for accuracy. (c) Illustrative examples of RCMCS similarity scores for three pairs of reactions. The maximum common subgraph inclusive of the RC is highlighted in green.

By separating our test data points into subsets based on their similarity to the training set, we support the first hypothesis that dissimilar data points make more challenging queries (Fig. 3). Nonetheless, all models maintain classification performance appreciably above chance over a substantial range of reaction similarity. The GNN models maintain above-chance performance even in the most extreme subset of dissimilar query reactions, unlike the Morgan fingerprint model. Moreover, the RC aggregation model distinguishes itself from the rest of the GNNs on this most difficult subset of data, maintaining nearly 78% accuracy. As an illustration of our reaction similarity metric, RCMCS, we show three reaction pairs with varying levels of similarity shown on the left of the chemical equations (Fig. 3c). We highlight in green the maximum common subgraph inclusive of the RC.

**Figure 4:**
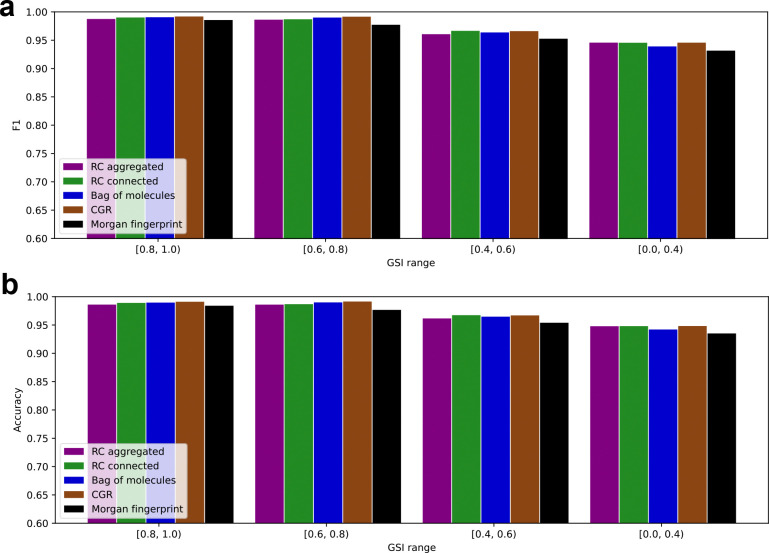
All models, including Morgan fingerprint benchmark, perform well on highly dissimilar enzymes. (a) Test set F1 score plotted against maximum GSI similarity between test data points and train data points. (b) Same as (a) for accuracy.

We repeated this analysis with a protein-based similarity metric, GSI. All models generalized extremely well to even the most dissimilar subset of enzymes (Fig. 4). This suggests that generalization to *de novo* reactions is more challenging than generalization to *de novo* enzymes.

### Embedding the three-hop neighborhood of the RC yields optimal performance

**Figure 5:**
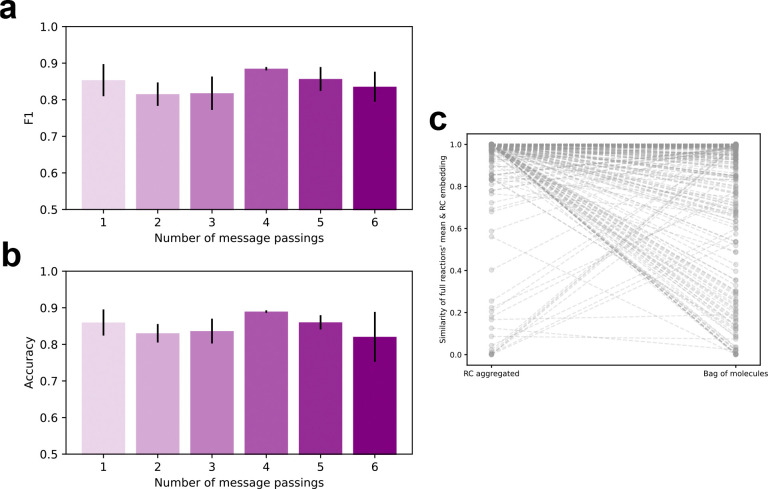
RC aggregated learns patterns relevant to reaction mechanisms. (a) F1 scores on validation data are shown for RC aggregation models using varied numbers of message passings corresponding to varied bond-wise distances from the RC. Error bars are standard deviation. (b) Same as (a) but with accuracy. (c) Shows the similarity of two vectors in the learned embedding space. The first vector is the mean of all reaction embeddings sharing the same RC. The second is the embedding of the RC itself. Each pair of points corresponds to a unique RC featured in the dataset embedded either with the RC aggregation or bag of molecules model.

As shown in Fig. 2, the RC aggregated model demonstrated outstanding generalization to unseen enzyme-reaction pairs, driven primarily by its performance on the most dissimilar subset of test pairs (Fig. 3). To account for this, we examined classification performance metrics on validation data for variants of the RC aggregated models varying only in the number of message passing steps during reaction embedding. These performance metrics cannot be interpreted as estimates of generalization error but can be compared to each other. Performance is optimal in the model variant with four message passing steps (Fig. 5 a and b). This model variant achieved the highest mean F1 and accuracy scores and the lowest variability in these same scores over three independent data splits. Four message passing steps imply that the reaction embedding vector depends only on substrate features within three bonds of the RC. It is interesting to compare this radius to those used in the design of reaction templates for computer-aided biosynthesis workflows. RetroRules use a radius of 3–4 bonds, aligned with our result here^19,40^. Others generate templates of variable size through a heuristic, but they nevertheless focus on substrate features within the local neighborhood of the RC^41,42^.

The choice to focus on local structure around the RC in enzymatic template extraction, also employed in this work, is informed by experience with enzymatic reaction mechanisms which tend to involve molecular structural features near the RC. We hypothesize that the chemistry-inspired form of graph augmentation used in the RC aggregated model introduces an inductive bias which is beneficial in the task of predicting enzyme-reaction pairs. In contrast, a less constrained model like bag of molecules may learn features that are not related to reaction mechanisms but are however still predictive in the enzyme-reaction prediction task.

As a test of this hypothesis, we compared the mean of all reaction embeddings of a common RC with the embedding of the RC itself. The RC alone is in a cheminformatics sense a valid, balanced reaction which we can encode as a SMILES string and pass to our model. We expected that an encoder that had learned patterns relevant to enzymatic reaction mechanisms would map reactions of a common RC near to the RC itself, each one differing slightly due to its specific characteristics. The mean of the full reaction vector embeddings should therefore be near the embedding of the RC itself. Using the RC aggregated encoder to embed reactions, we found that 94% of the time the full reactions’ vector mean had a similarity score of 0.8 or greater to the embedding of their shared RC. When using the bag of molecules to embed the reactions this dropped to 85%. Similarity scores were generated in the same manner as between a reaction and enzyme, which is also valid for this analysis since all inputs are mapped to the same vector space. Comparing RC by RC, the full reactions’ mean and RC embedding vector were closer in the majority (70%) of cases when encoded by the RC aggregated model compared to the bag of molecules model (Fig. 5c). This analysis is consistent with the idea that the RC aggregated model learns patterns that are more relevant to reaction mechanisms.

## Discussion

In this study we developed and compared models capable of successfully predicting whether a specific enzyme catalyzes a specific reaction. The RC aggregated exhibits the best generalization to *de novo* enzymes and reactions out of all models evaluated.

We demonstrated model generalization by extending methodologies routinely applied in protein property prediction tasks and multiclass classification. Following the example of prior work^24,32^, we used GSI to quantify pairwise similarity between enzymes. We extended this idea to pairwise reaction similarity with the RCMCS similarity metric. One can think of both GSI and RCMCS as methods that align graphs and score the alignments. In the case of amino acid sequences, graphs are linear. In the case of reactions, we have multiple disjoint subgraphs, the reactants and products. Maximum common subgraph with the extra condition to include the RC is an appropriate definition of alignment in this case. These similarity metrics served as inputs for our stratified similarity split method, which in turn enabled us to characterize model performance as a function of data point dissimilarity.

Like previous studies^24^, we observed that models performed extremely well over a wide range of enzyme dissimilarity. Estimates vary for the threshold on sequence identity after which protein function diverges. The most permissive is 40%^43^. Our results suggest all models we developed generalize well beyond the point at which memorizing sequence homology is effective. In part, we attribute this success to the representational power of protein language models^26^.

Generalization to dissimilar reactions is more difficult. The RC aggregated model performed best on this task. We attribute this to the key feature of this model, a chemistry-inspired form of graph augmentation which enables it to focus on patterns in reactant and product structures within a fixed window, optimally the 3-hop neighborhood, of the RC. We did not experiment with any form of semi-supervised technique for learning pre-trained reaction embeddings in contrast to the protein encoder. Future studies could investigate a form of masked language modeling appropriate for reaction graphs or related schemes.

The models developed here represent an important step forward in the formulation of enzymatic function prediction. Accommodating enzyme-reaction pairs as inputs removes ambiguity surrounding labels like EC number and multiplicity of reactions admitted by a given substrate. We share the belief that mapping reactions (especially previously unobserved reactions) to enzymes is a critical part of computer-aided biosynthesis^2^. Since our formulation of enzymatic function prediction is distinct from previous studies, we took care to design a series of benchmarks, each to address a specific question. Building up from the least featured, the Morgan fingerprint model lacks a differentiable message passing function and thus reveals the benefit of learning these transformations from data as the GNNs do. The bag of molecules model adds differentiable message passing but leaves out RC information, equivalently an all-atom mapping of the reaction. Both the RC connected and CGR models make use of all-atom mapping in different ways but globally aggregate information in contrast to the more narrowly focused RC aggregated model. A limitation of the RC aggregated, RC connected, and CGR models is the requirement of an all-atom mapping as an additional pre-processing step. There are tools to generate an all-atom mapping for a reaction including open-source and paid solutions^44^. One can also use minimal reaction templates to generate an all-atom mapping but are limited in this case to a set of known RCs^18,45^. Short of this, the bag of molecules model performs well in many conditions and does not require an all-atom mapping.

There are several use cases for a model such as the one developed here. In the field of computer-aided biosynthesis, it can map *de novo* reactions generated by network expansion tools^46,47^ to amino acid sequences. It can be used to annotate amino acid sequences without direct experimental activity including those with functions inferred from homology, which we purposefully excluded from our dataset. We anticipate, like other state-of-the-art protein function annotation tools, our model is not a replacement for experiments^48^, but it is conveniently formulated to guide experiments and can therefore be used in an iterative loop of prediction and validation. In addition to biotechnology, we believe our model can aid hypothesis generation in fundamental studies of evolvability and robustness by identifying secondary enzymatic activities that can compensate for genetic perturbation in sometimes elaborate ways, such as has been described in the pyridoxal 5’-phosphate synthesis pathway in *E. coli*^4,6^.

## Methods

### Data curation and pre-processing

Enzyme-reaction pairs were collected from the UniProt and Rhea databases^27,28^. We pulled all manually annotated UniProt (SwissProt) entries containing information in the ‘Catalytic activity’ field. Using the Rhea IDs listed in the Catalytic activity field of each UniProt entry, we gathered reaction information in the form of reaction SMILES.

To standardize the reaction SMILES, we removed stereochemistry, neutralized charges that vary depending on environmental conditions (e.g., protonating -OH groups), applied standard molecule normalization transformations, and canonicalized molecular SMILES. Standardization was carried out with cheminformatics library rdkit^49^. All transport reactions (e.g., between cellular components) were removed. We automatically generated RCs for each reaction by applying a set of minimal enzymatic reaction operators^18^ to reactants, alternately protecting all but one substructure matching the operator’s RC template.

From enzyme entries we gathered amino acid sequences and subsequently input them into ESM-1b to generate vector embeddings. We excluded proteins listed as subunits of a multi-unit catalytic complex, and those with ‘Level of Evidence’ classified by UniProt as ‘Inferred from Homology’, ‘Predicted’, or ‘Uncertain’.

### Model implementation

Models were built using Chemprop^35^, Lightning and PyTorch. Reaction encoders are implemented as custom classes subclassed from one of the three libraries depending on the encoder. For the protein encoder, we used the pre-trained ESM-1b model^26^. ESM-1b embeddings were linearly transformed by a matrix whose weights were learned from the data. The prediction head consists of taking a dot product of the reaction and enzyme embeddings and then applying the sigmoid function to the result.

All model parameters were learned using a binary cross entropy loss function with a positive weight parameter chosen through hyperparameter optimization.


l(x,y)=-[mpylog(σ(x))+(1-y)log(1-σ(x))]

Where m = negative multiple, p = positive multiplier, y = label, x = model output.

### Data splitting and negative sampling

In order to systematically evaluate model generalization error, we used a data splitting approach that we call stratified similarity split. Like normal stratified splitting, we arrange data across splits in a way that preserves representation of multiple categories. In stratified similarity split, a category is characterized by a range of maximum cross-split similarity score. For example, a data point assigned to a validation fold in the [0.4–0.6) category is between 0.4 and 0.6 similar to the nearest training fold data point. Stratified similarity split is applied in both the outer (test / training-validation) and inner splits (training / validation).

Stratified similarity split is achieved by first clustering the dataset according to a similarity metric up to a chosen upper bound on similarity, sampling clusters, relaxing the bound to the next value, and repeating the process up to the most permissive similarity bound. We carry out the process based on protein similarity, defined as GSI of locally aligned amino acid sequences, and reaction similarity, defined as the ratio of atoms in the RC-containing maximum common subgraph between reactions to the number of atoms in the larger of the two reactions.

In addition to the known positive enzyme-reaction pairs which we sourced from UniProt and Rhea, we assumed a number of negative pairs by randomly selecting unobserved enzyme-reaction pairs from our dataset. We sampled negatives in a one-to-one ratio with our positive pairs in test and validation folds and in a three-to-one ratio in training folds. The latter ratio was determined during hyperparameter optimization.

### Hyperparameter optimization

We selected values for several hyperparameters using a grid search and three-fold cross validation. These included model architecture parameters, e.g., number of message passings, dimension of hidden representation, training parameters, e.g., negative sampling ratio, number of epochs. A full list of optimized hyperparameters and their values appear in Table 2. In order to compare model architectures on the test set, we selected model architecture parameters that maximized performance in cross validation and standardized training parameters across model architectures to those which were most commonly optimal in cross-validation.

## Supplementary Material

Supplement 1

## Figures and Tables

**Table 1:** Key differences among reaction encoders.

	RC aggregated	RC connected	Bag of molecules	CGR	Morgan fingerprint
Learnable message passing function?	Yes	Yes	Yes	Yes	No
Aggregation function	Virtual node embedding	Mean of node embeddings	Mean of node embeddings	Mean of node embeddings	Absolute difference of reactant and product embedding sums
Virtual node?	Yes	Yes	No	No	No
Atom invariants	Chemprop v2	Chemprop v2	Chemprop v2	Chemprop v2	Daylight Atomic Invariants
Bond invariants	Chemprop default	Chemprop default	Chemprop default	Chemprop default	n/a

RC = reaction center; CGR = condensed graph of reaction

**Table 2:** Optimal hyperparameters used for each model architecture

	RC aggregated	RC connected	Bag of molecules	CGR	Morgan fingerprint
Training epochs	25	25	25	25	25
Negative multiple	3	3	3	3	3
Positive multiplier	3	3	3	3	3
Embedding dimension	300	300	300	300	300
# message passings	4	6	6	6 (RCMCS) / 4 (GSI)	n/a
ECFP radius	n/a	n/a	n/a	n/a	2
ECFP length	n/a	n/a	n/a	n/a	2048
Decision threshold	0.01 (RCMCS) / 0.12 (GSI)	0.01 (RCMCS) / 0.15 (GSI)	0.01 (RCMCS) / 0.26 (GSI)	0.01 (RCMCS) / 0.21 (GSI)	0.01 (RCMCS) / 0.05 (GSI)
