# Supplementary material for: RC-GNN: A predictive model of enzyme-reaction pairs
Source: bioRxiv. 2025 Jun 27:2025.06.22.660952. Preprint. [Version 1] doi: 10.1101/2025.06.22.660952 (PMC12262300; doi:10.1101/2025.06.22.660952)
Supplement: Supplement 1 [file NIHPP2025.06.22.660952v1-supplement-1.pdf]

bioRxiv preprint doi: https://doi.org/10.1101/2025.06.22.660952; this version posted June 27, 2025. The copyright holder for this preprint (which was not certified by peer review) is the author/funder, who has granted bioRxiv a license to display the preprint in perpetuity. It is made available under aCC-BY 4.0 International license.

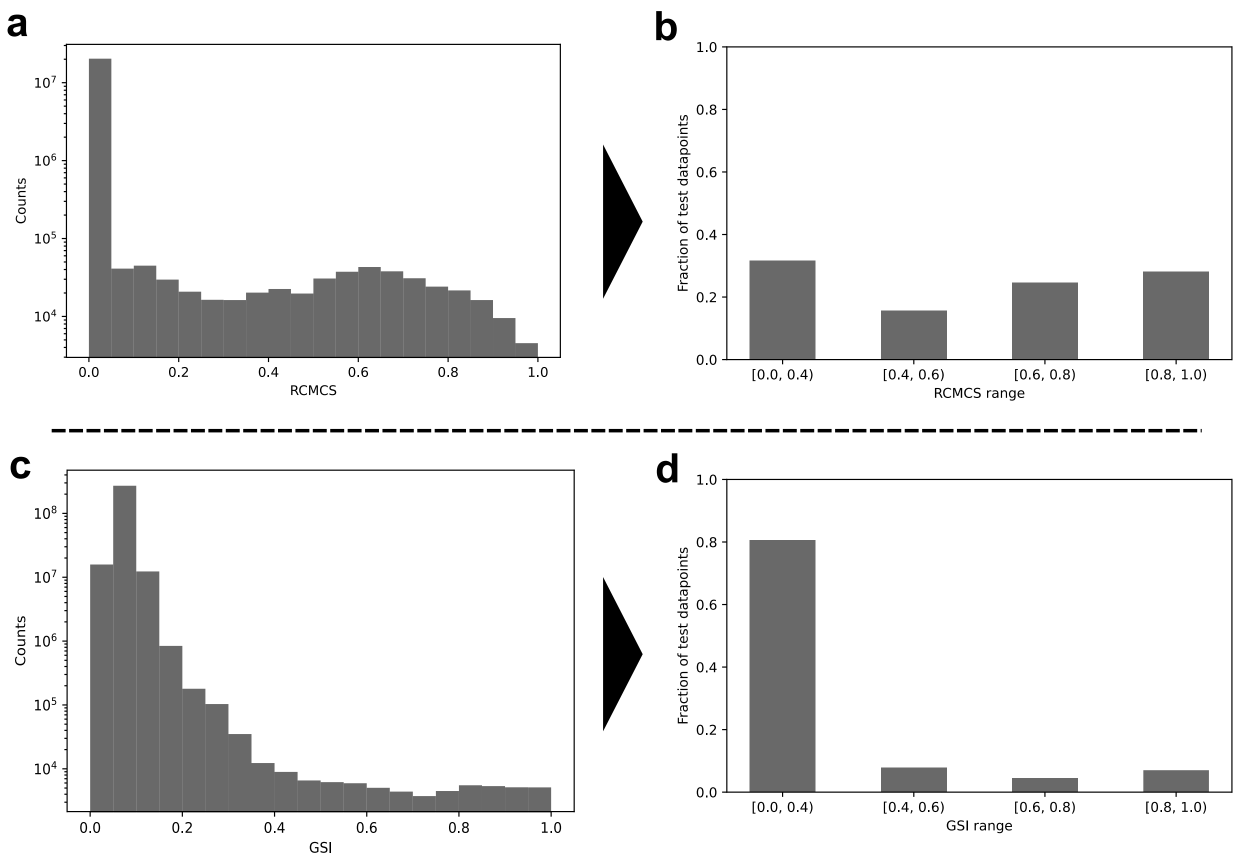

Figure S1 Stratified similarity split ensures the maximum similarity between test and train data points varies widely. (a) Histogram showing the background distribution of pairwise reaction similarities over the whole data set. (b) Stratified similarity split generates a test set approximately balanced in representation of similarity-defined subsets. (c) Same as (a) for pairwise protein similarity. (d) Stratified similarity split guarantees representation of similarity-defined subsets but is limited by the underlying distribution of pairwise similarities.

bioRxiv preprint doi: https://doi.org/10.1101/2025.06.22.660952; this version posted June 27, 2025. The copyright holder for this preprint (which was not certified by peer review) is the author/funder, who has granted bioRxiv a license to display the preprint in perpetuity. It is made available under aCC-BY 4.0 International license.

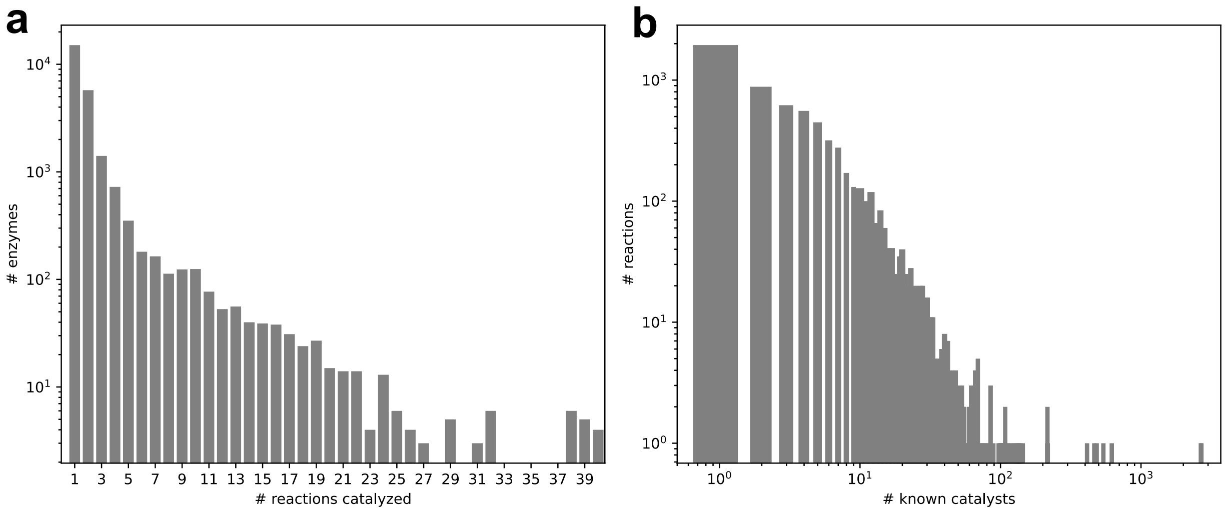

Figure S2 Dataset includes enzymes catalyzing multiple reactions and reactions catalyzed by multiple enzymes. (a) Histogram showing counts of unique enzymes catalyzing a given reaction (2.0 ± 2.6). (b) Histogram showing counts of unique reactions catalyzed by a given enzyme (7.6 ± 50. 4)

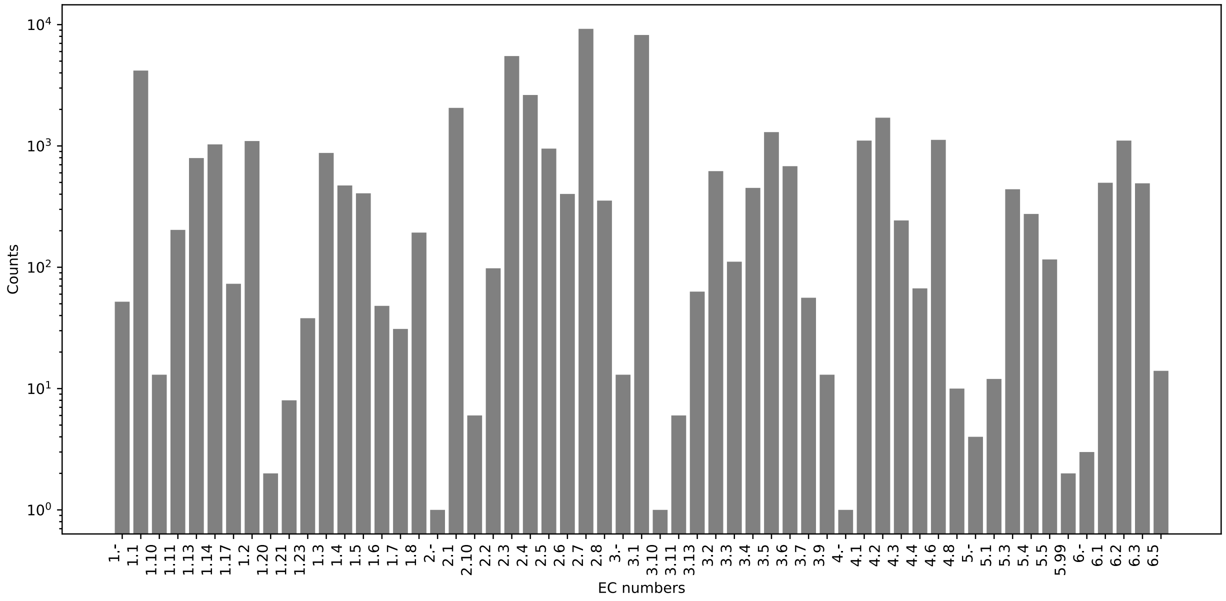

Figure S3 Dataset covers six major top-level EC numbers. Transport reactions were not included.
